# Clinical efficacy of acupuncture in the treatment of sciatica during the remission phase of lumbar disc herniation: A secondary analysis of a randomized controlled trial

**DOI:** 10.1097/MD.0000000000049151

**Published:** 2026-06-05

**Authors:** Fudong Shi, Zhiyi Wu, Shimin Zhang, Jinyan Yin, Yuzhang Liu, Zuoxu Li, Jiao Jin, Ning Liu, Guojun Wang, Haibao Wen, Chun Chen, Yadi Feng, Hai Lin

**Affiliations:** a Xiyuan Hospital, China Academy of Chinese Medical Sciences, Beijing, China; b Wangjing Hospital, China Academy of Chinese Medical Sciences, Beijing, China; c School of Traditional Chinese Medicine, Beijing University of Chinese Medicine, Beijing, China.

**Keywords:** acupuncture, lumbar disc herniation, remission phase, sciatica, subgroup analysis, traction

## Abstract

**Background:**

Sciatica caused by lumbar disc herniation (LDH) may persist during the remission phase and negatively affect patients’ quality of life. Although acupuncture is widely used for LDH, evidence regarding its efficacy specifically in remission-phase sciatica remains limited. This prespecified subgroup analysis aimed to compare the efficacy and safety of acupuncture versus traction in participants with remission-phase LDH-induced sciatica.

**Methods:**

Data were extracted from the acupuncture and traction groups of our multi-arm randomized controlled trial conducted at Wangjing Hospital between April 2022 and June 2024. We included participants with LDH-induced sciatica in the remission phase (VAS score ≥4 and <7). All participants received 3 weeks of treatment and were followed up for 16 weeks. The primary outcome was leg pain VAS (VAS-LP). Secondary outcomes included low back pain VAS (VAS-LBP), Oswestry Disability Index (ODI), and Japanese Orthopaedic Association (JOA) scores.

**Results:**

A total of 78 eligible participants (39 in the acupuncture group, 39 in the traction group) were included. At week 3 and week 4 follow-up, VAS-LP scores were significantly lower in the acupuncture group, with between-group differences of −0.63 cm (*P* = .019) and −0.86 cm (*P* = .017), respectively. At week 16, the difference was −0.51 cm (*P* > .05). After 3 weeks, JOA scores and JOA improvement rates were significantly higher in the acupuncture group. The excellent rate was 67.57% in the acupuncture group versus 47.22% in the traction group (*P* = .041). No significant differences were observed in VAS-LBP or ODI scores. Adverse events occurred in 8.1% of participants in the acupuncture group and were mild and transient.

**Conclusion:**

Acupuncture demonstrated superior short-term efficacy compared with traction in relieving leg pain during the remission phase of LDH and demonstrated a favorable safety profile.

## Graphical abstract

**Figure:**
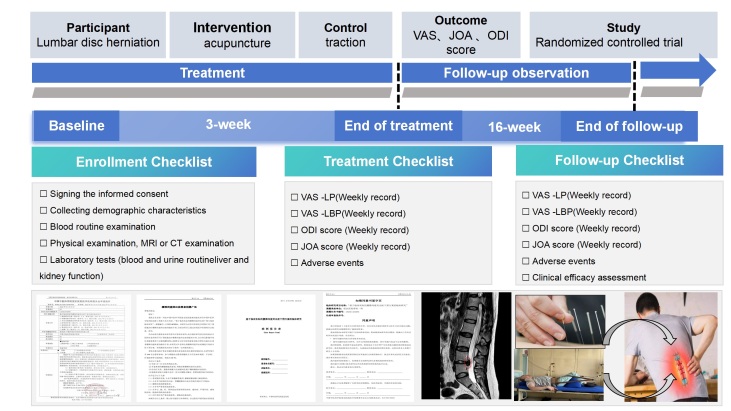

## 1. Introduction

Lumbar disc herniation (LDH) is a major cause of lower back pain and sciatica, most commonly occurring in people aged 25 to 55. The global annual incidence is 7.62%, and lifestyle changes have contributed to a rising incidence, particularly among younger populations.^[1]^ As it can sometimes hinder employment continuation or recovery, LDH has become a significant occupational health concern, imposing a heavy burden on both individuals and society. Based on clinical presentation and the Visual Analog Scale (VAS), LDH can be classified into 3 phases: the acute phase, remission phase, and recovery phase.^[2]^ Although nerve root inflammation and edema are relatively mild in the remission phase compared to the acute phase, residual symptoms such as low back pain and sciatica persist, with a risk of recurrence or worsening. This significantly affects participants’ daily functioning and work capacity. Additionally, during this phase, the disease enters a plateau with slow recovery, and conventional treatments often show limited efficacy, posing significant treatment challenges. For pain management, analgesics and surgery are commonly used to treat LDH-related pain.^[3]^ However, analgesics can cause side effects such as dizziness and nausea, and long-term use can lead to addiction and tolerance.^[4]^ Surgery is an invasive treatment, and only 10% to 20% of participants require it. Furthermore, some participants may experience residual pain, functional impairment, and psychological distress after surgery.^[5,6]^ Therefore, developing effective clinical treatment plans to alleviate low back pain and sciatica caused by LDH is crucial.

With evolving medical concepts and clinical needs, current guidelines and reviews are increasingly focusing on non-pharmacologicaltreatments for LDH, including exercise, spinal manipulation, and acupuncture.^[7,8]^ However, the challenge for both doctors and LDH participants remains in selecting conservative treatment methods that are more effective, have fewer side effects, and are more cost-efficient. Traditional Chinese Medicine (TCM) views LDH as being caused by the invasion of wind, cold, and dampness into the body, leading to poor circulation of Qi and blood, meridian blockage, and inadequate nourishment of muscles and bones. This leads to low back and leg pain, along with impaired lumbar function. Acupuncture, one of the oldest treatments in China, is based on TCM principles and is known to relax muscles, unblock meridians, and alleviate pain. It is an effective treatment for both acute and chronic low back and leg pain. Although studies have shown that acupuncture can alleviate inflammatory pain and sciatica,^[9]^ due to inconsistent guidelines, clinical opinions on whether to offer acupuncture treatment to LDH participants in the remission phase of sciatica vary.^[10-12]^ Therefore, the present study is a secondary analysis of a previously published randomized controlled trial,^[13]^ which compared the clinical efficacy of multiple non-pharmacological interventions in patients with LDH. Unlike the primary study, which evaluated overall treatment effects across a heterogeneous population, the current analysis specifically focuses on a clinically distinct subgroup, namely patients in the remission phase with sciatica.

## 2. Materials and methods

### 2.1. Study design

**Figure 1.**
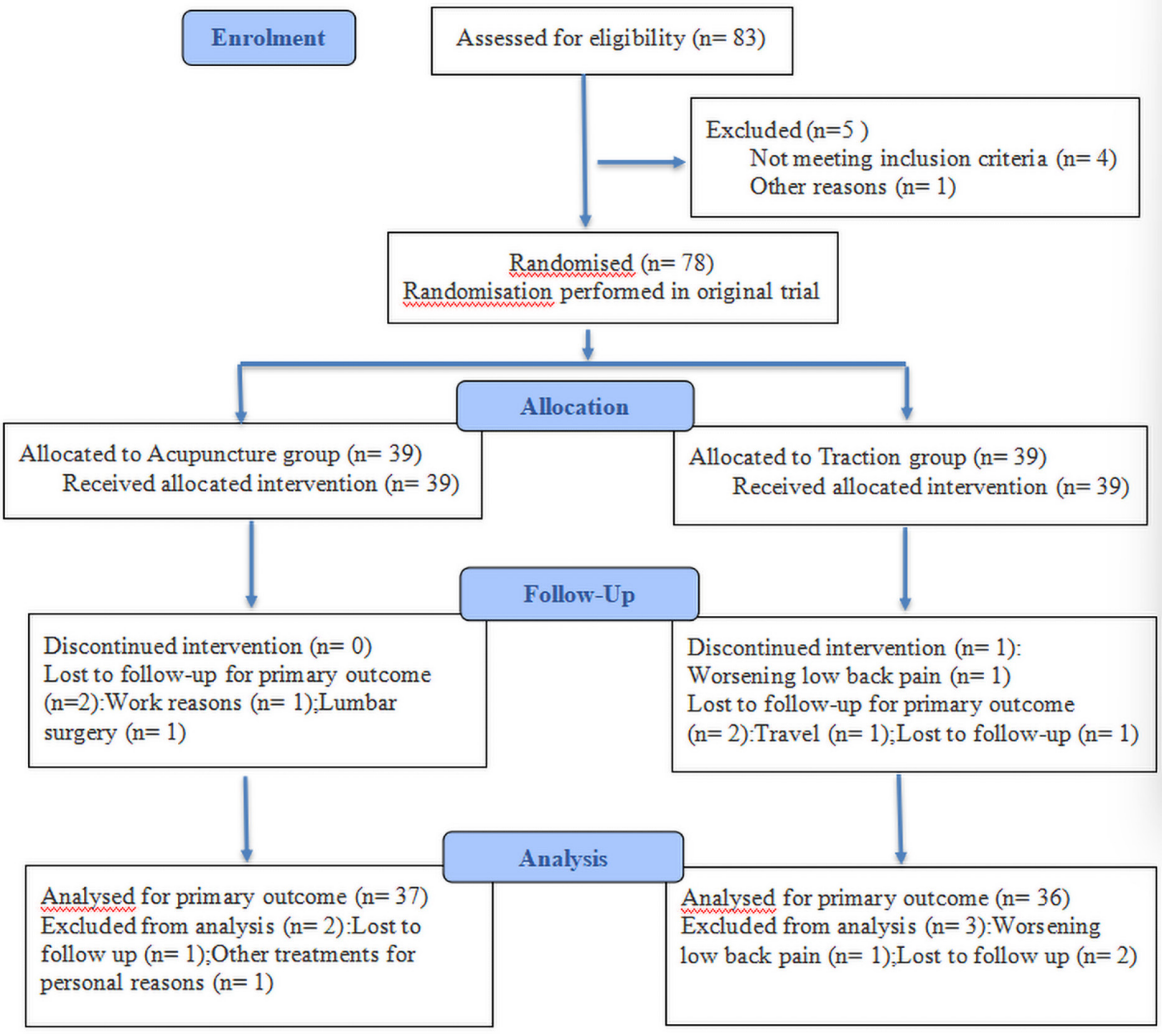
Consolidated standards of reporting trials (CONSORT) flow charts for enrollment and follow-up of all participants throughout the trial period. CONSORT = consolidated standards of reporting trials.

This study is a prespecified subgroup secondary analysis based on a previously published prospective, multi-arm, randomized, open-label, endpoint-blinded trial^[13]^ conducted at Wangjing Hospital of the China Academy of Chinese Medical Sciences between April 2022 and June 2024. The original trial recruited participants with LDH via hospital bulletins, and this analysis specifically focused on the subgroup of participants with LDH-induced sciatica in the remission phase (VAS score ≥4 and <7). A total of 78 eligible participants from the original trial’s acupuncture and traction groups were included in this analysis. All participants received treatment 3 times weekly for 3 weeks, followed by a 16-week follow-up period. Participants retained the right to withdraw their informed consent and discontinue participation at any stage of the study, consistent with the original trial protocol. The participant flow diagram for this subgroup analysis is presented in Figure 1.

### 2.2. Trial registration and ethics approval

The original trial protocol was approved by the Ethics Committee of Wangjing Hospital, China Academy of Chinese Medical Sciences (Approval No. WJEC-KT-2021-048-P003) and registered in the China Clinical Trial Registry on April 12, 2022 (Registration No. ChiCTR2200058598; URL: https://www.chictr.org.cn).

### 2.3. Participant recruitment

The diagnostic criteria for LDH in this analysis were consistent with the original trial, which adopted the North American Spine Society Clinical Guidelines.^[14]^ Eligibility screening for the original trial was performed by experienced clinicians, who confirmed participants’ eligibility based on clinical symptoms and lumbar MRI/CT imaging, and reviewed all inclusion and exclusion criteria.

For this subgroup analysis, we applied additional specific inclusion criteria to identify participants in the remission phase of sciatica: aged 18 to 60 years, regardless of gender; presented with typical low back and leg pain, diagnosed with LDH-induced sciatica, and had a VAS score ≥4 and <7; completed the original trial’s informed consent process and adhered to treatment and follow-up requirements.

The exclusion criteria were identical to those of the original trial, including: obvious osteophyte formation complicated by severe lumbar stenosis, spondylolisthesis, or spondylolysis; presence of cauda equina syndrome, conus medullaris syndrome, or other absolute surgical indications; severe osteoporosis; active skin injury, infection, or dermatological disorders at the treatment site; severe cardiovascular, endocrine, or psychiatric diseases; pregnancy, breastfeeding, or planned pregnancy during the study period; and history of spinal surgery. Participants who met any exclusion criterion were excluded from this analysis within 24 hours of screening.

### 2.4. Randomization

The randomization procedure was fully implemented in accordance with the original trial protocol. An independent statistician not involved in participant recruitment, treatment delivery, or outcome assessment generated the random allocation sequence using SAS 9.4 software (SAS Institute Inc.). A research assistant independent of the study team printed the randomization cards and placed them in sequentially numbered, sealed, opaque envelopes.

Trained clinicians enrolled participants and assigned sequential numbers from 01 to 78 based on enrollment order. An independent research coordinator opened the corresponding numbered envelope to assign each participant to a treatment group. Random numbers were non-reassignable once allocated, and participants who discontinued the study were not replaced. This analysis included all participants originally randomized to the acupuncture and traction groups who met the subgroup eligibility criteria.

### 2.5. Blinding

Consistent with the original trial design, blinding of participants, treating physicians, and study investigators was not feasible due to the inherent nature of acupuncture and traction interventions. To mitigate potential bias in outcome assessment, all research staff responsible for data collection, endpoint measurement, and statistical analysis remained blinded to group allocation throughout the study period.

### 2.6. Interventions

In the acupuncture group, participants were instructed to lie prone with their lower back exposed. Disposable Huatuo needles (Tianjin-produced Hanyi brand, 0.25 mm × 50 mm) were used for the treatment, while larger needles (0.25 mm × 75 mm) were used for the Huantiao point (GB30). After disinfecting the acupuncture points, needles were inserted at bilateral Jiaji points (EX-B2), Dachangshu (BL25), Shenshu (BL23), and Huantiao (GB30); and on the healthy side at Houxi (SI3), Weizhong (BL40), and Chengshan (BL57). All needles were inserted at a 90° angle, with even supplementation and draining techniques. Once a deqi sensation (acupuncture sensation) or radiating pain was achieved, the needles were retained for 30 minutes. After removal, sterile cotton swabs were used to apply pressure to stop any bleeding.

In the traction group, the initial traction force was calibrated to 40% of each participant’s body weight and titrated upward to a maximum of 50% based on their comfort level. For elderly participants or those with poor physical tolerance, the starting force was reduced to 30% and incrementally raised to no more than 40%. Traction parameters were dynamically adjusted according to individual response: higher forces were delivered for shorter time intervals, whereas lower forces were sustained for longer durations. Each traction session was conducted for 20 to 30 minutes using a Sanjie OL-2000 traction bed (Okiki Medical Co., Ltd., Japan).

All interventions were delivered 3 times weekly on alternate days for a total of 3 consecutive weeks.

### 2.7. Outcome measures

The primary outcome was improvement in sciatica, which was measured by changes in leg pain VAS (VAS-LP) scores from baseline at 1 week, 3 weeks, 4-week follow-up, and 16-week follow-up. The VAS is a 0 to 10 cm scale, where 0 cm represents no pain and 10 cm represents unbearable pain. Participants marked a point on the scale that reflected their average leg pain intensity over the past week.

The secondary outcomes included assessment of low back pain using VAS (VAS-LBP) at the 4 predefined time points, with the same measurement method as VAS-LP. Additionally, we evaluated improvements in lumbar function by measuring changes in Oswestry Disability Index (ODI) scores from baseline at the same time points. The ODI is a self-administered questionnaire with 10 items measuring functional disability (we excluded the sexual activity item to avoid patient discomfort). Each item offers 6 statements scored 0 to 5, describing various possible situations in daily life. Participants selected the statement that best described their situation over the past week, with lower scores indicating better condition. Third, we assessed the recovery of lumbar nerve function by evaluating the improvement rate of Japanese Orthopedic Association (JOA) scores, also measured at the 4 aforementioned time points. The JOA scoring system includes subjective symptoms (low back pain, leg pain, and gait), clinical signs (straight leg raise, sensory disturbance, motor dysfunction), degree of daily activity limitation, and bladder function, covering a total of 14 items with a maximum score of 29. The improvement rate formula is: (Posttreatment score − Pretreatment score)/(29 − Pretreatment score) × 100%. Clinical improvement was categorized into 4 levels based on the percentage of symptom improvement, as described in the primary study: poor (<25%), fair (25–49%), good (50–74%), and excellent (75–100%). Higher percentages indicated greater recovery of lumbar nerve function.

Adverse events were recorded, assessed, treated, and their incidence was calculated throughout the study.

### 2.8. Sample size calculation

The sample size for this analysis was derived from the acupuncture and traction arms of a previously published randomized controlled trial. No additional sample size calculation was performed for the present secondary analysis.

To assess whether the available sample size was adequate to detect a clinically meaningful difference, we performed a post hoc power analysis using PASS 11.0 software (NCSS, LLC). Using a 2-sided significance level of α = 0.05 and a predefined clinically relevant between-group difference of 0.5 cm in VAS-LP score change at 3 weeks, the analysis indicated that the 73 evaluable participants provided sufficient statistical power to detect this effect size.

### 2.9. Safety indicators and adverse event management

The safety monitoring and adverse event (AE) management protocol was identical to that used in our original randomized controlled trial. A standardized AE reporting system was implemented throughout the study period. All adverse reactions that occurred during treatment and follow-up were recorded in real time, with detailed documentation of onset time, severity grade, duration, and specific intervention measures. The study team analyzed the potential etiology of each AE and assessed its causal relationship with the study interventions.

If participants experienced palpitations, dizziness, blurred vision, or excessive diaphoresis during acupuncture, the procedure was immediately terminated. Participants were positioned supine to rest and provided with warm drinking water. For participants who reported exacerbated low back or leg pain after traction therapy, the traction force and treatment duration were dynamically adjusted based on individual tolerance and clinical response.

### 2.10. Statistical analysis

Data were analyzed using IBM SPSS Statistics, version 25.0 (International Business Machines Corporation, China). A 2-sided test with a 95% confidence interval and a significance level of 0.05 was applied. For baseline characteristics, continuous variables were tested using either an independent *t* test or a nonparametric test depending on normality and homogeneity of variance. Categorical variables were tested using the chi-square test.

For outcome measures, leg pain VAS, low back pain VAS, ODI, and JOA improvement scores were analyzed for between-group differences using independent *t* tests or nonparametric tests based on normality and homogeneity of variance. Paired *t* tests or nonparametric tests were used for comparisons with baseline data depending on normality and variance.

## 3. Results

This prespecified subgroup analysis included participants from the acupuncture and traction arms of our previously published randomized controlled trial. A total of 83 participants underwent eligibility screening for this remission-phase sciatica study, of whom 5 were excluded due to abnormal imaging findings or failure to meet the subgroup-specific inclusion criteria.

Seventy-eight eligible participants were included in the final analysis cohort, all of whom had been previously randomized to either the acupuncture group (n = 39) or the traction group (n = 39). During the 3-week treatment period, 1 participant in the traction group discontinued due to aggravated low back pain. During the 16-week follow-up period, 2 participants in the acupuncture group withdrew (1 for work-related reasons and 1 due to subsequent lumbar surgery), and 2 participants in the traction group withdrew (1 for travel and 1 was lost to follow-up).

Overall, 37 participants in the acupuncture group and 36 in the traction group completed the entire follow-up. A total of 73 participants were included in the final per-protocol statistical analysis. The participant flow diagram for this subgroup analysis is presented in Figure 1.

### 3.1. Baseline characteristics of all participants

Detailed demographic and clinical characteristics of the study participants are summarized in Table 1. The mean age of all participants was 44.62 ± 10.48 years, with no participants exceeding 60 years of age. Most participants had a normal body mass index (BMI) ranging from 18 to 24 kg/m^2^, and all vital signs (body temperature, pulse rate, and respiratory rate) were within normal physiological limits at baseline.

**Table 1:** Baseline characteristics of the participants.

Characteristic	All participants (n = 78)	Ac group (n = 39)	Tr group (n = 39)	Effect size	*P* value
Age, mean (SD)	44.62 ± 10.48	45.10 ± 10.05	42.92 ± 10.99	0.625	.534
Female, No. (%)	45 (61.64)	22 (59.46)	23 (63.89)	0.530	.467
BMI, mean (SD), kg/m^2^	22.78 ± 4.03	22.18 ± 4.23	23.48 ± 3.32	0.098	.922
Temperature, mean (SD), °C	36.45 ± 0.29	36.33 ± 0.34	36.33 ± 0.33	0.620	.537
Pulse, mean (SD), cpm	71.54 ± 5.62	71.69 ± 6.28	71.39 ± 6.68	1.345	.183
Respiration, mean (SD), cpm	18.34 ± 3.02	18.52 ± 2.38	18.13 ± 3.31	0.753	.454
Course of disease, mean (SD), d	1028.64 ± 748.71	1039.45 ± 946.33	1012.15 ± 942.75	0.808	.422
Time of onset, mean (SD), d	46.32 ± 39.13	48.37 ± 34.18	44.22 ± 39.58	1.067	.785
Primary outcomes, mean ± SD, *P*					
** **VAS-LP score	6.04 ± 0.52	5.97 ± 0.45	6.1 ± 0.58	−1.021	.311
Secondary outcomes, mean ± SD, *P*					
** **VAS-LBP score	6.05 ± 0.74	6.03 ± 0.74	6.05 ± 0.76	−0.117	.907
** **ODI score	25.74 ± 4.27	25.81 ± 3.53	25.67 ± 4.97	0.143	.887
** **JOA score	12.6 ± 2.93	12.73 ± 2.72	12.47 ± 3.18	0.373	.710

BMI = body mass index, JOA = Japanese Orthopedic Association, ODI = Oswestry Disability Index, SD = standard deviation, VAS = visual analog scale (1 to 10 m), VAS-LBP = Visual Analog Scale for Low Back Pain.

### 3.2. Primary outcome

**Figure 2.**
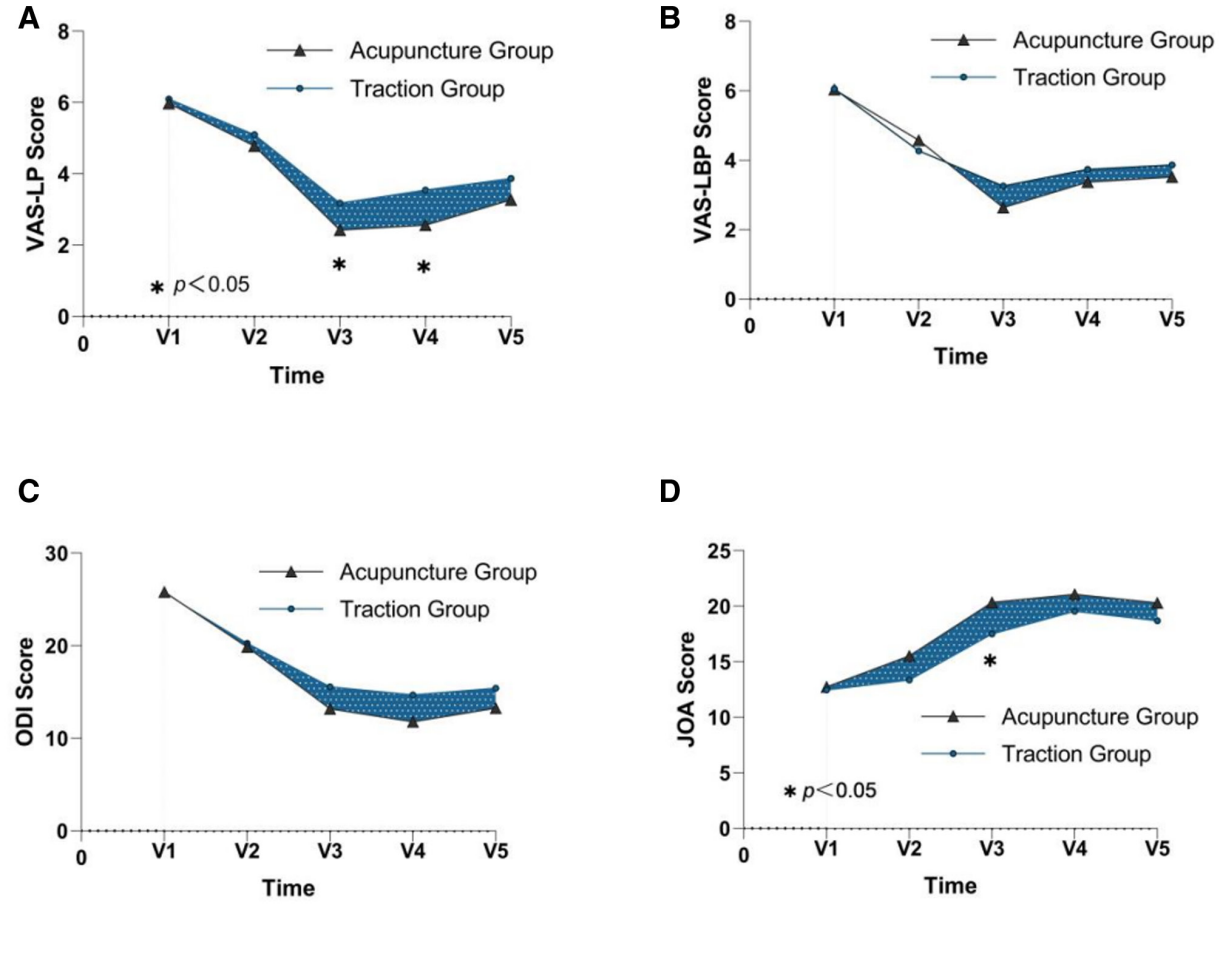
(A–D) Comparison of VAS-LP scores, VAS-LBP scores, ODI scores, and JOA scores over time during treatment and follow-up in both groups. Lower VAS-LP, VAS-LBP, and ODI scores indicate clinical improvement, while higher JOA scores indicate clinical improvement. Visits: baseline (V1), 1 week of treatment (V2), 3 weeks of treatment (V3), 4-week follow-up (V4), 16-week follow-up (V5). JOA = Japanese Orthopaedic Association, ODI = Oswestry Disability Index, VAS-LBP = Visual Analog Scale for Low Back Pain, VAS-LP = Visual Analog Scale for Leg Pain.

Visual Analog Scale for Leg Pain (VAS-LP) is a primary measure of sciatica in LDH. As shown in Table 2, the change in VAS-LP from baseline in the acupuncture group was −3.55 cm (95% CI = −4.01, −3.10) after 3 weeks of treatment and −2.71 cm (95% CI = −3.24, −2.17) at the 16-week follow-up. In the traction group, the changes were −2.93 cm (95% CI = −3.19, −2.66) after 3 weeks and −2.23 cm (95% CI = −2.74, −1.72) at 16 weeks. Both groups showed a gradual reduction in leg pain VAS scores, with a greater reduction in the acupuncture group. The trend of VAS-LP reduction in both groups was shown in Figure 2A. At the 3-week and 4-week follow-up, significant differences were observed between the groups, with a difference of −0.63 cm (95% CI = −1.15, −0.11, *P* = .019) at 3 weeks and −0.86 cm (95% CI = −1.56, −0.15, *P* = .017) at 4 weeks. At the 16-week follow-up, the between-group difference was −0.51 cm (95% CI = −1.20, 0.25), which exceeded 0.5 cm but was not statistically significant.

**Table 2:** Primary and secondary outcomes: change from baseline.

Outcome	Acupuncture group (N = 37), mean (95% CI)	Traction group (N = 36), mean (95% CI)	Between-group difference, mean (95% CI)	*P* value
Primary outcomes				
** **Average VAS in leg pain†,‡				
** **1 wk	−1.19 (−1.45, −0.93)*	−1.00 (−1.28, −0.73)*	−0.19 (−0.56, 0.18)	.318
** **3 wk	−3.55 (−4.01, −3.10)*	−2.93 (−3.19, −2.66)*	−0.63 (−1.15, −0.11)	**.019**
** **4 wk follow up	−3.41 (−3.90, −2.93)*	−2.56 (−3.08, −2.03)*	−0.86 (−1.56, −0.15)	**.017**
** **16 wk follow up	−2.74 (−3.24, −2.17)*	−2.23 (−2.74, −1.72)*	−0.51 (−1.20, 0.25)	.195
Secondary outcomes				
** **Average VAS in low back pain†,‡				
** **1 wk	−1.46 (−1.79, −1.13)*	−1.79 (−2.18, −1.40)*	0.33 (−0.17, 0.83)	.191
** **3 wk	−3.40 (−3.89, −2.91)*	−2.80 (−3.22, −2.38)*	0.60 (−1.24, 0.04)	.066
** **4 wk follow up	−2.66 (−3.42, −1.91)*	−2.32 (−2.79, −1.86)*	−0.34 (−1.22, 0.54)	.441
** **16 wk follow up	−2.52 (−3.45, −1.58)*	−2.20 (−2.72, −1.67)*	−0.32 (−1.38, 0.74)	.548
** **Mean ODI‡				
** **1 wk	−5.95 (−7.69, −4.2)*	−5.42 (−8.1, −2.73)*	−0.53 (−3.66, 2.60)	.737
** **3 wk	−12.62 (−14.39, −10.86)*	−10.11 (−12.3, −7.92)*	−2.51 (−5.26, 0.24)	.073
** **4 wk follow up	−14.03 (−16.05, −12.01)*	−10.97 (−13.4, −8.55)*	−3.06 (−6.15, 0.04)	.053
** **16 wk follow up	−12.54 (−14.73, −10.35)*	−10.25 (−12.83, −7.67)*	−2.29 (−5.61, 1.03)	.174
** **Mean JOA				
** **1 wk	2.03 (1.1, 2.96)*	0.89 (−0.3, 2.08)	1.14 (−0.34, 2.62)	.129
** **3 wk	7.62 (6.35, 8.89)*	5.03 (3.76, 6.3)*	2.59 (0.83, 4.36)	**.005**
** **4 wk follow up	8.35 (6.90, 9.80)*	7.08 (5.52, 8.65)*	1.27 (−0.83, 3.36)	.231
** **16 wk follow up	7.57 (6.19, 8.94)*	6.22 (4.6, 7.85)*	1.35 (−0.74, 3.43)	.203
** **Mean JOA IR (%)				
** **1 wk	15.60 (10.71, 20.68)*	10.29 (1.92, 18.67)*	5.40 (−4.12, 14.91)	.262
** **3 wk	50.20 (44.08, 56.33)*	41.21 (35.61, 46.81)*	9.00 (0.83, 17.16)	**.031**
** **4 wk follow up	52.10 (44.46, 59.73)*	42.58 (34.76, 50.40)*	9.52 (−1.22, 20.26)	.082
** **16 wk follow up	44.60 (37.41, 51.79)*	36.24 (27.52, 44.97)*	8.36 (−2.72, 19.44)	.137

Bold values indicate statistically significant differences.

CI **= **confidence interval, JOA **=** Japanese Orthopedic Association, JOA IR = Japanese Orthopaedic Association Improvement Rate, ODI **=** Oswestry Disability Index, VAS **=** visual analog scale (1 to 10 m).

† Leg/low back pain intensity in the past week.

‡ Lower value indicates better status; higher value indicates better status.

* *P* < .05 when compared with baseline.

### 3.3. Secondary outcome

#### 3.3.1. Visual Analog Scale for Low Back Pain (VAS-LBP)

VAS-LBP is the primary measure for low back pain. As shown in Table 2, the average VAS-LBP scores in both groups gradually decreased over the 3-week treatment and 16-week follow-up period, with a greater reduction in the acupuncture group. The trend in VAS-LBP reduction in both groups was shown in Figure 2B. The changes in VAS-LBP from baseline in the acupuncture group were −3.40 cm (95% CI = −3.89, −2.91) after 3 weeks of treatment and −2.20 cm (95% CI = −2.72, −1.67) at 16 weeks. In the traction group, the changes were −2.80 cm (95% CI = −3.22, −2.38) after 3 weeks and −0.32 cm (95% CI = −1.38, 0.74) at 16 weeks. Additionally, the between-group difference in VAS-LBP scores at 3 weeks was −0.60 cm (95% CI = −1.24, .04, *P* = .066), which exceeded 0.5 cm but was not statistically significant.

#### 3.3.2. Oswestry Disability Index (ODI)

ODI is a commonly used scale to assess lumbar function. As shown in Table 2, there were no significant differences between the 2 groups during the 3-week treatment and 16-week follow-up periods. However, both the acupuncture and traction groups showed a gradual reduction in ODI scores compared to baseline, with the greatest reduction observed at the 4-week follow-up: −14.03 (95% CI = −16.05, −12.01) for the acupuncture group and −10.97 (95% CI = −13.4, −8.55) for the traction group, indicating a greater reduction in the acupuncture group. The trend of ODI reduction was shown in Figure 2C.

#### 3.3.3. Japanese Orthopedic Association (JOA) Score (JOA)

The JOA score is a key measure for evaluating symptom improvement and nerve function recovery. As shown in Table 2, JOA scores improved over time during treatment. At the end of 3 weeks of treatment and at the 16-week follow-up, the acupuncture group had increases of 7.62 (95% CI = 6.35, 8.89) and 7.57 (95% CI = 6.19, 8.94), respectively, while the traction group showed increases of 5.03 (95% CI = 3.76, 6.30) and 6.22 (95% CI = 4.60, 7.85). At 3 weeks, the between-group difference was statistically significant, with a difference of 2.59 (95% CI = 0.83, 4.36, *P* = .005). The trend of increasing JOA scores in both groups was shown in Figure 2D.

At the end of 3 weeks of treatment and at the 16-week follow-up, the improvement rate (IR) of JOA in the acupuncture group was 50.20% (95% CI = 44.08, 56.33) and 44.60% (95% CI = 37.41, 51.79), respectively, compared to 42.58% (95% CI = 34.76, 50.40) and 36.24% (95% CI = 27.52, 44.97) in the traction group. At 3 weeks, the JOA IR between the groups showed a significant difference, with a group difference of 9.00% (95% CI = 0.83, 17.16).

**Figure 3.**
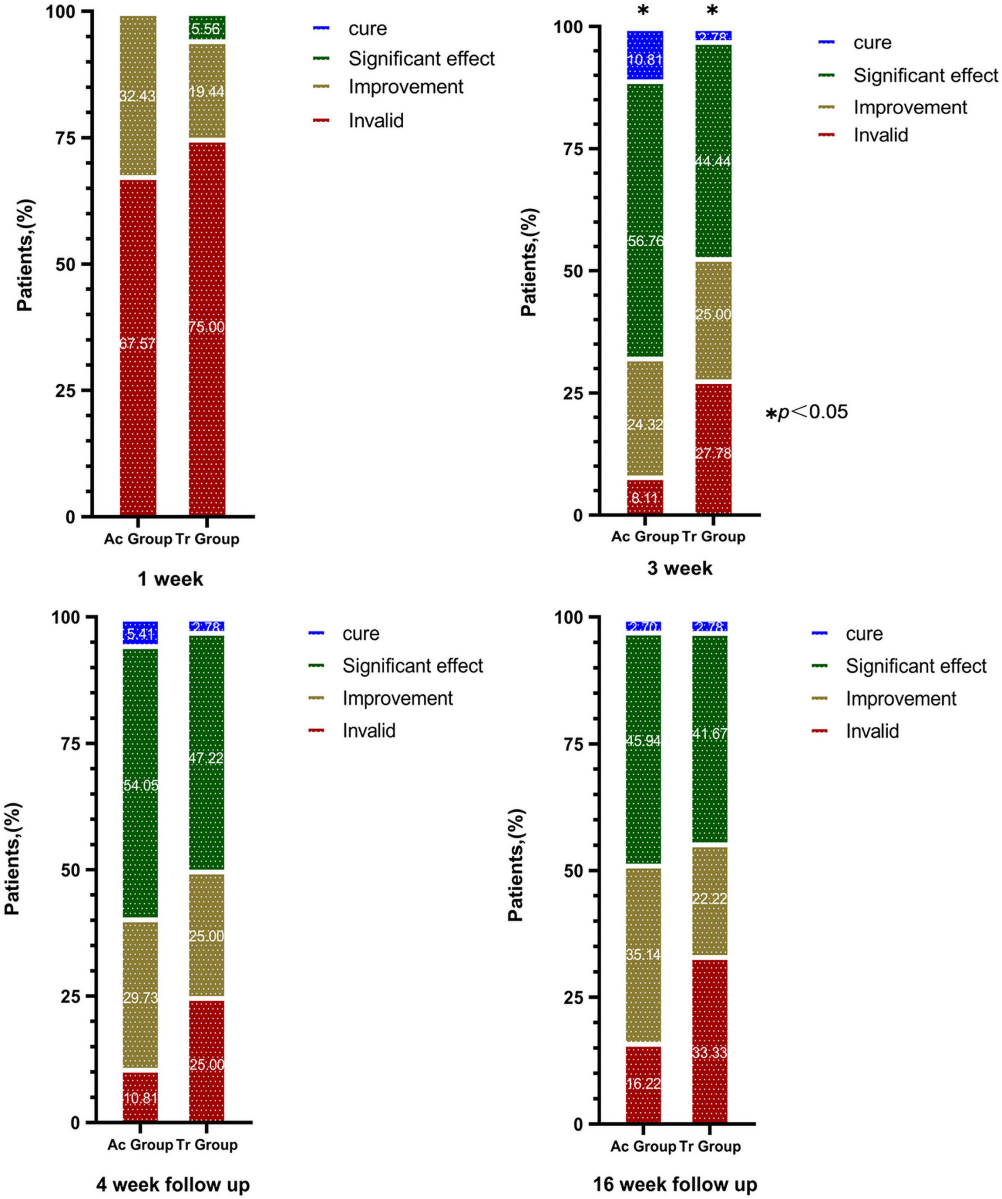
Comparison of clinical efficacy assessments during treatment and follow-up in both groups.

After 3 weeks of treatment, clinical efficacy based on JOA IR was evaluated. In the acupuncture group, 4 participants (10.81%) were cured, 21 participants (56.76%) showed significant improvement, 9 participants (24.32%) showed improvement, and 3 participants (8.11%) showed no effect. In the traction group, 1 patient (2.78%) was cured, 16 participants (44.44%) showed significant improvement, 9 participants (25.00%) showed improvement, and 10 participants (27.78%) showed no effect. This difference was statistically significant (χ^2^ = 8.254, *P* = .041). The clinical efficacy based on JOA IR was illustrated in Figure 3.

### 3.4. Adverse events

No serious adverse events occurred during the study, and all adverse events were mild or transient. In the acupuncture group, 5 participants experienced adverse events (3/37, 8.1%), including 1 case of subcutaneous hematoma after acupuncture (1/37, 2.7%) and 2 cases of fainting (palpitations, dizziness, cold sweats) after acupuncture (2/37, 5.4%). Symptoms resolved within 10 minutes after the participants were placed in a supine position and given warm water. In the traction group, 2 participants reported mild worsening of low back and leg pain after treatment (2/36, 5.6%). No participants in either group used analgesics during the study.

## 4. Discussion

This study provides additional evidence regarding the clinical efficacy of acupuncture in patients with lumbar disc herniation during the remission phase with sciatica. The findings suggest that acupuncture may offer beneficial effects in pain relief and functional recovery in this specific subgroup. Importantly, the present study differs from our previously published trial in several key aspects.^[13]^ While the original study evaluated the comparative efficacy of multiple non-pharmacological interventions in a general LDH population, the current analysis focuses specifically on patients in the remission phase with persistent sciatica, representing a more homogeneous and clinically relevant subgroup. In addition, the research objective of this study is more focused, aiming to evaluate the efficacy of acupuncture in a targeted clinical context rather than comparing multiple interventions simultaneously. These differences underscore the distinct clinical value of the present analysis, as it provides more precise evidence to guide individualized treatment strategies.

Acupuncture treatment for LDH is grounded in Traditional Chinese Medicine’s understanding of the lumbar and leg pain caused by LDH. Acupoints are selected along the meridians, focusing on adjusting the flow of Qi and blood in the Du and Bladder meridians, which run through the lumbar and back, to balance Yin and Yang.^[15]^ Located between the Du Meridian and Bladder Meridian, Jiaji points (EX-B2) serve as the primary acupoints for managing LDH due to their critical function in balancing these 2 meridians. Previous studies have demonstrated that acupuncture at the Jiaji points stimulates the skin and muscle tissues innervated by the posterior branches of the spinal nerves, promoting local blood circulation in the lumbar region.^[16]^ Additionally, acupuncture at Jiaji may stimulate the corresponding spinal nerve roots, activating endogenous analgesic substances, reducing nerve root edema, and resolving sterile inflammation, thereby exerting anti-inflammatory and analgesic effects.^[17]^ The Dachangshu (BL25) point regulates Qi and blood in the lumbar region and improves local blood circulation.^[18]^ The Shenshu (BL23) point, located on the back, serves as the kidney’s Shu point and has a kidney-tonifying effect, promoting bone marrow production. Acupuncture at Shenshu plays a crucial role in nourishing the kidney and strengthening the bones, thus helping to alleviate muscle spasms in the lumbar region.^[19]^

Moreover, this study is based on the theory of “balancing Yin and Yang” and “seeking harmony through shared Qi.” Acupuncture on the contralateral limb utilizes the physiological characteristics of meridian pathways connecting the upper and lower body, as well as the left and right sides, to harmonize Yin and Yang, unblock meridians, and relieve pain. We selected the contralateral Houxi (SI3) point, as it unblocks the Du meridian, regulates the Qi of the Du meridian, and harmonizes the Bladder meridian, effectively relieving pain.^[20]^ We also chose the contralateral Weizhong (BL40) point, which is the He point of the Bladder meridian, known for its ability to unblock the meridian and stimulate organ Qi, effectively treating lower limb pain. In Traditional Chinese Medicine, the Weizhong point is also considered the main point for treating low back pain, as reflected in the saying: “For back pain, seek Weizhong.” Acupuncture at Weizhong increases the pain threshold, thereby relieving lumbar and leg pain.^[21]^

This study involving 73 participants with LDH in the remission phase of sciatica showed that, compared to traction, acupuncture may have a better short-term effect in reducing leg pain in these participants. At 3 weeks and 4 weeks follow-up, the acupuncture group showed decreases in VAS-LP scores of −3.55 (−4.01, −3.10) and −3.41 (−3.90, −2.93), respectively, while the traction group showed decreases of −2.93 (−3.19, −2.66) and −2.56 (−3.08, −2.03). The between-group differences were −0.63 cm (95% CI = −1.15, −0.11, *P* = .019) and −0.86 cm (95% CI = −1.56, −0.15, *P* = .017). According to the American College of Physicians (ACP) clinical practice guidelines,^[22]^ the magnitude of treatment effects is defined as follows: a small effect is a VAS between-group difference of 0.5 to 1.0 points, a moderate effect is 1.0 to 2.0 points, and a large effect is >2.0 points. Acupuncture achieved a clinically significant effect.

No statistically significant difference was observed between the 2 groups in VAS-LP or ODI scores. However, at week 3, the difference in VAS-LBP between the 2 groups was −0.60 cm (95% CI = −1.24, 0.04), indicating a small effect size in favor of the acupuncture group for relieving low back pain, which has clinical significance.

This study further demonstrated that JOA scores in both the acupuncture and traction groups increased over time. Previous research has indicated that the minimum clinically important difference (MCID) for JOA scores is 2.5 points.^[23]^ At week 3, the between-group difference in favor of the acupuncture group was 2.59 (95% CI = 0.83, 4.36), reaching the MCID. Moreover, clinical improvement in the acupuncture group reached 50.20%, significantly higher than the 42.58% observed in the traction group. However, there were no statistically significant or clinically meaningful differences between the groups in week 1 or during the follow-up period.

The results of this study are generally consistent with current evidence, which suggests that acupuncture can effectively relieve sciatica caused by LDH.^[24-26]^ However, a treatment duration of at least 3 weeks or longer may be required to achieve satisfactory clinical outcomes. Some studies have questioned acupuncture’s effectiveness in relieving joint adhesions and restoring joint mobility.^[27,28]^ Our study did not demonstrate any significant difference in lumbar function between the 2 groups. We postulated that although acupuncture can alleviate clinical symptoms in participants with LDH by inducing relaxation of tense and spasmodic paraspinal muscles, it possesses inherent therapeutic limitations when used as monotherapy. It primarily affects local muscle spasm and stress concentration, which may gradually alter joint dysfunction, so improvements in local lumbar function may appear more slowly.^[29,30]^ In addition, traction, a widely used physical therapy for LDH, has shown significant clinical effectiveness.^[31,32]^ However, this study indicates that acupuncture’s role in improving lumbar function remains unclear due to the influence of treatment protocols and time. Further research is warranted to clarify this.

## 5. Limitations

Several limitations of this study should be acknowledged. First, this was a prespecified subgroup analysis of a multi-arm randomized controlled trial, and the sample size was not prospectively calculated for this head-to-head comparison. While post hoc power analysis was adequate, results should be interpreted cautiously and cannot be generalized broadly. Second, no placebo or blank control group was included, and the relatively short treatment and follow-up periods may have introduced statistical biases. Larger samples and longer follow-up would clarify long-term efficacy. Third, blinding of treating clinicians and participants was not feasible due to the nature of the interventions. Although research staff involved in data collection and analysis were blinded, residual subjective bias cannot be completely ruled out. Finally, multiple comparisons across time points and outcomes may have increased the risk of type I error, and all outcome measures were subjective self-reported scales. Future studies should incorporate objective assessments such as electromyography to confirm these findings.

## 6. Conclusions

Overall, acupuncture may provide better short-term efficacy than traction in relieving leg pain and promoting symptom improvement and nerve function recovery in participants with sciatica during the remission phase of LDH, without any serious adverse events. This study offers evidence supporting the use of acupuncture for sciatica in the remission phase of LDH, but further research with larger sample sizes, longer treatment periods, and extended follow-up durations is needed to verify these findings.

## Acknowledgments

We are grateful to thank all editors/reviewers for their helpful comments and valuable suggestions.

## Author contributions

**Conceptualization:** Fudong Shi, Shimin Zhang, Yuzhang Liu, Jiao Jin.

**Data curation:** Fudong Shi, Zhiyi Wu, Shimin Zhang, Jinyan Yin, Jiao Jin, Guojun Wang, Haibao Wen, Chun Chen.

**Investigation:** Jinyan Yin, Jiao Jin, Chun Chen, Yadi Feng, Hai Lin.

**Methodology:** Zuoxu Li, Ning Liu, Guojun Wang, Haibao Wen.

**Project administration:** Fudong Shi, Shimin Zhang, Hai Lin.

**Supervision:** Zhiyi Wu, Shimin Zhang, Ning Liu, Guojun Wang.

**Validation:** Ning Liu.

**Writing – original draft:** Fudong Shi.

**Writing – review & editing:** Shimin Zhang.
